# Are the determinants of vertebral endplate changes and severe disc degeneration in the lumbar spine the same? A magnetic resonance imaging study in middle-aged male workers

**DOI:** 10.1186/1471-2474-9-51

**Published:** 2008-04-16

**Authors:** Mari Kuisma, Jaro Karppinen, Marianne Haapea, Jaakko Niinimäki, Risto Ojala, Markku Heliövaara, Raija Korpelainen, Kaisu Kaikkonen, Simo Taimela, Antero Natri, Osmo Tervonen

**Affiliations:** 1 Department of Diagnostic Radiology, Oulu University Hospital, Oulu, Finland; 2 Department of Physical and Rehabilitation Medicine, Oulu University Hospital, Oulu, Finland; 3 Public Health Institute, Helsinki, Finland; 4 Department of Sports Medicine, Oulu Deaconess Institute, Oulu, Finland; 5 Department of Public Health, University of Helsinki, Finland; 6 Department of Orthopaedics, Tampere University Hospital, Tampere, Finland; 7 Musculoskeletal Centre, Finnish Institute of Occupational Health, Oulu, Finland; 8 ORTON Orthopedic Hospital, Helsinki, Finland; 9 Department of Public Health and General Practice, University of Oulu, Finland

## Abstract

**Background:**

Modic changes are bone marrow lesions visible in magnetic resonance imaging (MRI), and they are assumed to be associated with symptomatic intervertebral disc disease, especially changes located at L5-S1. Only limited information exists about the determinants of Modic changes. The objective of this study was to evaluate the determinants of vertebral endplate (Modic) changes, and whether they are similar for Modic changes and severe disc degeneration focusing on L5-S1 level.

**Methods:**

228 middle-aged male workers (159 train engineers and 69 sedentary factory workers) from northern Finland underwent sagittal T1- and T2-weighted MRI. Modic changes and disc degeneration were analyzed from the scans. The participants responded to a questionnaire including items of occupational history and lifestyle factors. Logistic regression analysis was used to evaluate the associations between selected determinants (age, lifetime exercise, weight-related factors, fat percentage, smoking, alcohol use, lifetime whole-body vibration) and Modic type I and II changes, and severe disc degeneration (= grade V on Pfirrmann's classification).

**Results:**

The prevalences of the Modic changes and severe disc degeneration were similar in the occupational groups. Age was significantly associated with all degenerative changes. In the age-adjusted analyses, only weight-related determinants (BMI, waist circumference) were associated with type II changes. Exposure to whole-body vibration, besides age, was the only significant determinant for severe disc degeneration. In the multivariate model, BMI was associated with type II changes at L5-S1 (OR 2.75 per one SD = 3 unit increment in BMI), and vibration exposure with severe disc degeneration at L5-S1 (OR 1.08 per one SD = 11-year increment in vibration exposure).

**Conclusion:**

Besides age, weight-related factors seem important in the pathogenesis of Modic changes, whereas whole-body vibration was the only significant determinant of severe disc degeneration.

## Background

Vertebral endplate (Modic) changes are bone marrow and endplate lesions visible in magnetic resonance imaging (MRI). They are shown to be associated with degenerative intervertebral disc disease [1-3]. Three different types have been described [2,3]. Type I lesions (low T1 and high T2 signals) are assumed to indicate an ongoing active degenerative process. Type II lesions (high T1 and T2 signals) are thought to manifest a more stable and chronic degeneration. Type III lesions (low T1 and T2 signals) are associated with subchondral bone sclerosis.

It has been shown that a multitude of factors determine the risk of disc degeneration [4-12]. Several studies have shown Modic changes as an indicator of symptomatic disc degeneration [13-18]. However, there is only limited information about the determinants of Modic changes. In addition to age [2,19,20], body weight [19], heavy physical work for more than 10 years [14], smoking more than 20 cigarettes a day [14] and male gender [19] have shown associations with Modic changes.

Modic changes are interesting as an association between Modic changes and low back symptoms has been shown recently in population-based cohorts [13,14,21]. The purpose of this study was to evaluate the determinants of Modic changes, and whether Modic changes and disc degeneration share common etiological factors in middle-aged male workers. Our main focus was on the L5-S1 level, because a significant association between Modic changes and low back pain intensity during the past week was found in this same population only at L5-S1 [21].

## Methods

### Study population

The study population consisted of 228 Caucasian males with a mean age of 47 years (range: 36–56 years) at the time of enrolment. Train engineers (n = 159) worked for the Finnish state railways, and had a mean of 21 years (range: 5–31 years) of exposure to whole-body vibration. They were all full-time train drivers with approximately five-hour daily exposure to whole-body vibration composed of both vertical and horizontal components. They were all from northern Finland, which ensured that they had been operating the same kinds of locomotives and had similar exposure to vibration. Their work contained no significant exposure to manual material handling.

The other occupational group consisted of 69 paper mill and chemical factory workers with a mostly sedentary job and no vibration exposure. All study members participated on a voluntary basis and received no compensation. Approval from Institutional Review Board and signed, informed consent from each patient were obtained before MR imaging. The study was approved by the Ethics Committee of Oulu University Hospital.

### Assessments

Before MR imaging, the participants received a self-administered questionnaire containing items on occupational history, such as lifetime exposure to whole-body vibration, and lifestyle factors. A specially trained nurse processed the answers.

The questionnaire requested a complete lifetime occupational history including the following elements: job titles and duties, type of activity required by the job, and duration of employment in each job. To estimate the exposure to whole body vibration, the subjects were asked to report whether during the day they spent time in a motorized vehicle and, if this was the case, to state the approximate mean number of daily driving hours.

Smoking was reported as years smoked and the average number of cigarettes smoked per day. For smoking exposure, the number of pack years smoked was calculated with the following formula: Pack years = Average daily cigarette consumption divided by 20 multiplied by years smoked.

Alcohol use was reported as number of portions (glasses of wine (12 cl), measures of spirits (4 cl), or bottles of beer (0.33 liter), each corresponding to 12 g of alcohol) consumed during the week before completion of the questionnaire. Alcohol consumption was divided a priori into six categories (never, seldom, 2 to 3 times a month, 3 to 4 times a week, once or twice a week and daily). In the statistical analyses, alcohol consumption was classified as "Once a month or less", "2 to 3 times a month", "1 to 4 times a week" and "More than 4 times a week".

To assess the frequency and intensity of leisure time physical activity, a modified Paffenbarger questionnaire was used [22,23]. The subjects were asked to recall their participation in activities during three time periods in their life span, corresponding to the ages of 15, 30, and their current age. Exercise was categorized as mild, moderate, or strenuous, and the options were given the scores 1, 2 and 3, respectively. Subjects reported the highest level of exercise performed for at least 15 minutes at a time, at least three times a week at each evaluated time point. The lifetime exercise score thus represents the sum of the scores, ranging from 3 to 9, at the evaluated ages.

Body weight (kg), height (m), waist circumference (cm), and body fat percentage (%) were measured by a nurse. Body Mass Index (BMI) was calculated as weight (kg) divided by height squared (m^2^). Percentage of fat and lean mass was assessed using bioimpedance equipment (Bodystat 1500, Bodystat Ltd., Douglas, Isle of Man, UK).

### Magnetic Resonance Imaging (MRI)

Magnetic resonance imaging was performed using a 1.5-T unit (Signa, General Electric, Milwaukee, WI) with Phased Array CTL Spine Coil (USA Instruments, Aurora, OH). The imaging protocol consisted of sagittal T1-weighted (1809/18 [repetition time msec/echo time msec]) fluid-attenuated inversion recovery (FLAIR) and sagittal T2-weighted (3960/116) fast spin-echo (FSE) imaging of the entire lumbar spine. The inversion recovery time for T1-weighted images was 660 msec, and the number of excitations for both T1- and T2-weighted images was four. Echo train length (ETL) for T1-weighted images was eight, and for T2-weighted sagittal images 29. The image matrix was 448 × 192 for T1-weighted images and 448 × 224 for T2-weighted sagittal images. Field of view (FOV) for sagittal images was 28 × 28 cm. Slice thickness was 4 mm and interslice gap 1 mm.

### Image Analysis

**Figure 1:**
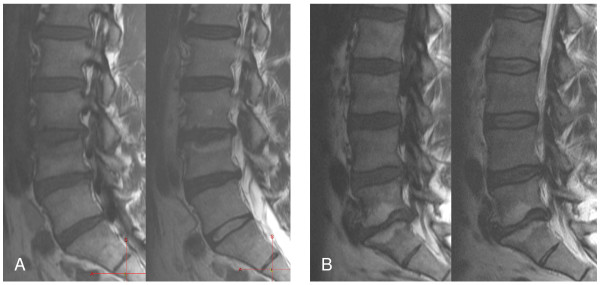
**A**: **MR images of the lumbar spine in a 40-year-old paper mill worker demonstrate Modic type I change (low T1 and high T2 signals) at both endplates of L3-L4.B**: MR images of the lumbar spine in a 49-year-old train engineer demonstrate Modic type II change (high T1 and T2 signals) at both endplates of L5-S1.

Modic changes and intervertebral disc degeneration were evaluated from MRI scans. Modic changes were assessed independently by two radiologists (MK, RO), and a consensus was negotiated in case of disagreement. Both readers were blinded to subjects' clinical status. Classification of Modic changes was carried out at a workstation on the basis of T1- and T2-weighted images based on the five midsagittal planes. Both the upper and the lower endplates at each disc level were graded separately into types MI, MII, or MIII, as previously defined (Figure 1), and mixed Modic types I/II or II/III [2,18]. In the analyses, types I and I/II were grouped together, as all lesions containing type I change are assumed to indicate a more active process. Similarly, types II and II/III were grouped together, as they are thought to manifest a more stable and chronic process. Signal intensity changes associated with Schmorl's nodes, or tiny spots of signal intensity change in the bone marrow adjacent to the vertebral corners were not recorded. An analysis of inter-reader reliability showed an almost perfect agreement in the evaluation of Modic types [21]. The degree of disc degeneration was classified by one reader (JN) based on the five midsagittal T2-weighted images according to Pfirrmann's grading system, which showed good repeatability in the original work [24]. In this classification, disc degeneration is graded into five classes. Grade five in the classification indicates severe degeneration; the structure of the disc is inhomogeneous, signal intensity is hypointense, the distinction between nucleus and annulus is lost, and the disc space is collapsed.

### Statistical Analysis

Logistic regression analysis was used to evaluate the association between selected determinants and 1) type of Modic changes and 2) severe disc degeneration. Odds ratios (ORs) with 95% confidence intervals (CI) were calculated per an increment of one standard deviation unit for each continuous explanatory variable. Analyses were carried out for all lumbar levels combined and separately for changes located at L5-S1.

All subjects were included in the analyses of Modic changes, i.e. either type I or type II at any level (N = 228). Subjects who had both type I and type II change at the same or separate level (N = 23) were excluded when analyzing determinants of individual Modic types. In the analyses of Modic changes located at L5-S1, subjects who did not have Modic change at L5-S1 but had any Modic change at the upper levels were excluded (N = 42). The reference group in the analyses of Modic changes consisted of 100 subjects without any Modic changes, whereas in the analyses of severe disc degeneration the reference group consisted of 163 subjects with at most grade IV degeneration. The age-adjusted analyses were first done separately for each single determinant. Based on these analyses, multivariate analysis was conducted. For the multivariate analysis, only the significant determinants (for either Modic changes or severe disc degeneration) were included. The data were analyzed with the SPSS for Windows software, version 14.0.

## Results

### Description of the study population

Characteristics of the study population are presented in Table 1. The two occupational groups were similar in height and current smoking status. Train engineers had higher lifetime leisure exercise activity score (p = 0.02), whereas sedentary workers tended to be heavier (p = 0.08) and had significantly higher BMI and fat percentage (p = 0.02 and 0.03, respectively), and larger waist circumference (P = 0.004). Sedentary workers consumed alcohol more frequently compared to train engineers (p < 0.001).

**Table 1:** Distribution of selected determinants among train engineers and sedentary factory workers.

	Train engineers N = 159		Factory workers N = 69	
	
	Mean (SD)	Range	Mean (SD)	Range
Vibration (years)	21.3 (4.7)	5–31	-	-
Age (years)	46.0 (3.2)	38–53	47.8 (4.5)	36–56
BMI (kg/m^2^)	25.8 (2.7)	18.3–33.9	26.9 (3.7)	21.1–41.5
Waist circumference (cm)	91.9 (8.7)	74–121	95.8 (10.5)	73–133
Fat percentage (%)	21.4 (4.9)	11–40	23.4 (8.5)	6–46
Lifetime exercise ^1^	7	3–9	6	3–9
Current smoking ^2^				
No	125 (80%)		52 (75%)	
Yes	31 (20%)		17 (25%)	
Smoking among current smokers (pack years)	23.4 (12.1)	1–61	16.7 (11.5)	1–35
Alcohol consumption ^2^				
Once a month or less	41 (27%)		7 (10%)	
2 to 3 times a month	55 (36%)		17 (25%)	
1 to 4 times a week	57 (37%)		27 (39%)	
More than 4 times a week	0 (0%)		18 (26%)	

^1 ^Data as Median.^2 ^Data as N (%).

### Prevalence of Modic changes and disc degeneration

Out of 228 subjects studied, 128 (56%) were found to have Modic change at one or more levels. Thirty-three (15%) had exclusively type I change, while 72 (32%) had exclusively type II change at one or more levels. Twenty-three (10%) subjects had both type I and type II changes either at separate levels or at the same disc level (i.e. different type on cranial and caudal endplates). Eighty-six (38%) subjects had Modic change at L5-S1; 25 (11%) had type I, 58 (25%) type II, and three subjects had both type I and type II change at L5-S1.

**Figure 2:**
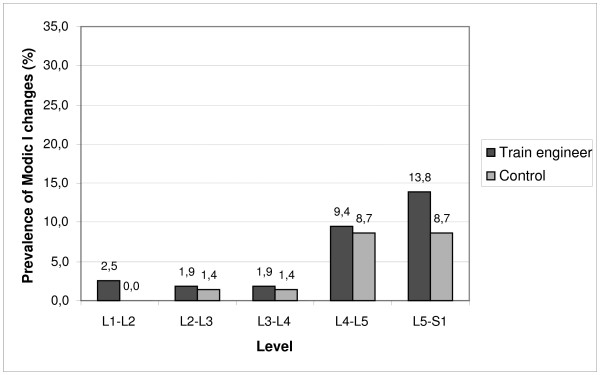
Prevalence of Modic type I changes in discs from L1-2 to L5-S1 among train engineers and factory workers.

**Figure 3:**
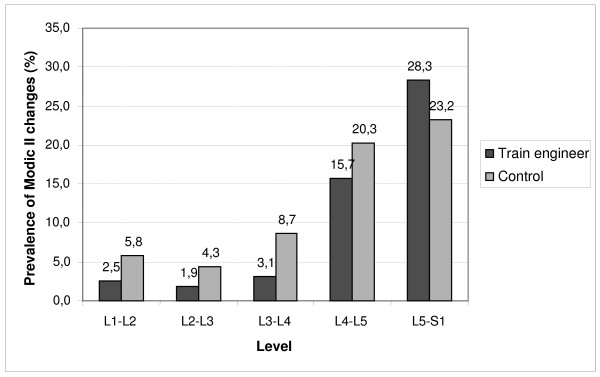
Prevalence of Modic type II changes from L1-2 to L5-S1 among train engineers and factory workers.

When the occupational groups were analyzed separately, 87 (55%) of the train engineers and 41 (59%) of the factory workers were found to have Modic changes (P = 0.562). The corresponding figures for Modic I and II changes are shown in Table 2. The occurrence of Modic changes increased to the caudal direction in both occupational groups. 80% of Modic changes being located at the two lowest levels (Figures 2, 3).

**Table 2:** Number (proportion) of MRI findings among train engineers and sedentary factory workers.

	Train engineers N = 159		Factory workers N = 69		
		
	N	%	N	%	P value ^1^
Modic changes					
Type I	26	27%	7	20%	0.502
Type II	45	39%	27	49%	0.246
Pfirrmann grade V degeneration	48	30%	17	24%	0.237

^1 ^P value from chi-square test.

Out of 228 subjects studied, 65 (28%) were found to have severe (grade-five) disc degeneration at one or more levels (Table 2). Forty-nine (21%) subjects had severe disc degeneration at L5-S1. Thirty-nine (24%) of them were train engineers and 10 (14%) sedentary controls (p = 0.059).

As the prevalence of Modic changes and severe disc degeneration was comparable in both occupational groups, they were combined for the logistic regression analyses of selected determinants.

### Determinants of Modic changes

Table 3 shows the association between the selected determinants and Modic changes for the whole study population. Modic changes were age-dependent (Tables 3 and 4). In the age-adjusted analyses (Table 3), none of the analyzed determinants were associated with Modic type I changes, whereas both weight-related factors (BMI and waist circumference) were associated with type II changes at L5-S1 when type II changes were included exclusively (ORs 1.42 and 1.48, respectively). Similar results were obtained when all lumbar levels were combined. Occupation (train engineers vs. factory workers), duration of exposure to whole-body vibration (years), body fat percentage or alcohol consumption were not significantly associated with Modic type II changes. There was a significant association between lifetime leisure exercise activity and all Modic changes (type I and II combined) in the univariate analyses (data not shown). However, when types I and II were analyzed separately, no significant associations remained (Table 3).

**Table 3:** Associations between the selected determinants and Modic Type I changes, Modic Type II changes and severe disc degeneration, presented for all vertebral levels and for the L5-S1 level separately. Age-adjusted odds ratios (OR) per one standard deviation (SD) are calculated for continuous variables. Subjects with both types are not included in the analyses of Types I and II.

		Modic Type I				Modic Type II				Severe disc degeneration ^b^			
		
		All levels^1 ^N = 133		L5-S1^2 ^N = 121		All levels^3 ^N = 172		L5-S1^4 ^N = 149		All levels^5 ^N = 228		L5-S1^6 ^N = 212	
		
	SD	OR	95% CI	OR	95% CI	OR	95% CI	OR	95% CI	OR	95% CI	OR	95% CI
Age	4 years	1.13	0.73–1.58	1.35	0.84–1.93	**1.56**	1.21–1.95	**1.54**	1.14–1.96	**1.46**	1.13–1.82	**1.43**	1.07–1.82
Train engineers vs. factory workers	NA	1.51	0.58–3.91	1.87	0.56–6.24	0.82	0.41–1.63	1.44	0.62–3.32	1.80	0.89–3.61	**2.48**	1.08–5.67
Vibration exposure	11 years	1.00	0.59–1.41	1.05	0.58–1.55	0.95	0.64–1.26	1.20	0.83–1.58	1.27	0.97–1.59	**1.41**	1.05–1.78
Body mass index	3 kg/m^2^	1.14	0.77–1.54	1.08	0.67–1.54	**1.36**	1.02–1.75	**1.42**	1.03–1.86	1.17	0.87–1.49	1.10	0.78–1.45
Waist circumference	9 cm	1.10	0.73–1.49	1.02	0.59–1.47	**1.42**	1.08–1.77	**1.48**	1.11–1.89	1.12	0.82–1.42	1.07	0.76–1.40
Fat percentage	6 %	1.10	0.69–1.54	1.01	0.94–1.10	1.08	0.77–1.42	1.11	0.71–1.53	1.15	0.85–1.46	1.16	0.82–1.51
Lifetime exercise ^a^	2 units	1.24	0.80–1.78	1.59	0.94–2.49	1.20	0.86–1.60	1.30	0.89–1.80	1.13	0.82–1.49	1.14	0.80–1.55
Smoking	10 pack years	1.41	0.99–1.84	1.43	0.94–1.94	1.17	0.85–1.51	1.23	0.89–1.59	1.14	0.87–1.43	1.10	0.80–1.42
Regular vs. no alcohol use	NA	0.71	0.30–1.68	0.77	0.27–2.21	1.09	0.53–2.24	1.00	0.45–2.22	1.25	0.63–2.49	1.42	0.65–3.10

All levels: ^1 ^33 subjects with only Modic I changes, ^3 ^72 with only Modic II changes and ^5 ^65 with severe disc degeneration.

Level L5-S1: ^2 ^21 subjects with only Modic I changes, ^4 ^49 with only Modic II changes and ^6 ^49 with severe disc degeneration.

Subjects with type I changes were excluded from the analyses of type II changes. Similarly, subjects with type II changes were excluded from the analyses of type I changes. The reference group in the analyses of Modic changes consisted of 100 subjects with no Modic changes, and in case of severe disc degeneration of 163 subjects with at most grade IV degeneration.

^a ^Lifetime leisure exercise activity is a score from 1 to 9; 1–4 mild, 5–7 moderate and 8–9 high activity.

^b ^Severe disc generation = Pfirrmann grade V

**Table 4:** Multivariate models of the predictors of Modic Type II changes and severe disc degeneration, presented for all vertebral levels and for the L5-S1 level separately. Odds ratios (OR) per one standard deviation (SD) are calculated for continuous variables. Subjects with both types are not included in the analyses of Type II.

		Modic Type II				Severe disc degeneration ^a^			
		
		All levels^1 ^N = 172		L5-S1^2 ^N = 149		All levels^3 ^N = 228		L5-S1^4 ^N = 212	
		
	SD	OR	95% CI	OR	95% CI	OR	95% CI	OR	95% CI
Age	4 years	**1.55**	1.19–1.94	**1.60**	1.18–2.05	**1.52**	1.17–1.90	**1.51**	1.11–1.94
BMI	3 kg/m^2^	**2.29**	1.05–3.67	**2.75**	1.31–4.37	1.70	0.64–2.87	1.54	0.39–2.83
Vibration exposure	11 years	1.00	0.95–1.06	1.05	0.99–1.12	**1.05**	1.00–1.11	**1.08**	1.01–1.14

All levels: ^1 ^72 with only Modic II changes and ^3 ^65 with severe disc degeneration.

Level L5-S1: ^2 ^49 with only Modic II changes and ^4 ^49 with severe disc degeneration.

Subjects with type I changes were excluded from the analyses of type II changes.

The reference group in the analyses of Modic changes consisted of 100 subjects with no Modic changes, and in case of severe disc degeneration of 163 subjects with at most grade IV degeneration.

^a ^Severe disc generation = Pfirrmann grade V

In the multivariate model, in addition to age, BMI was associated significantly with type II changes at L5-S1 (OR 2.75; 95% CI 1.31–4.37). Waist circumference was also significant (OR 1.18 [per one SD = each 3 cm increase in the measure]; 95% CI 1.05–1.32) but due to collinearity of these two weight-related factors they could not be included in the model simultaneously. Exposure to whole-body vibration was of borderline significance at L5-S1 (OR 1.05; 95% CI 0.99–1.12). Similar results were obtained when all lumbar levels were combined (Table 4).

### Determinants of severe disc degeneration

Severe disc degeneration was age-dependent (Tables 3 and 4). The duration of exposure to whole-body vibration associated with higher prevalence of grade-five disc degeneration at L5-S1 (OR 1.41; 95% CI 1.05–1.78). Similarly, being a train engineer (vs. sedentary factory work) associated with disc degeneration at L5-S1 (OR 2.48; 95% CI 1.08–5.67). Whole-body vibration did not associate with severe disc degeneration when all lumbar levels were combined (Table 3). Mechanical factors (BMI, waist circumference, lifetime exercise), fat percentage, smoking, or alcohol consumption were not significantly associated with severe disc degeneration (Table 3). In the multivariate model, in addition to age, exposure to whole-body vibration associated with severe disc degeneration at L5-S1 (OR 1.08 [per one SD = each 11-year increment in vibration exposure]; 95% CI 1.01–1.14; Table 4).

## Discussion

Our study showed that weight-related factors contribute to the likelihood of Modic changes but not to severe disc degeneration. Whole-body vibration was associated with severe disc degeneration at L5-S1, whereas it was of borderline significance in case of type II Modic changes at L5-S1. To the authors' knowledge, only one population-based study [14] has previously focused on the determinants of Modic changes. The authors found that heavy physical work for more than 10 years and heavy smoking of more than 20 cigarettes a day were associated with Modic changes [14].

In a recent population-based study among 40-year-old Danish men and women, the prevalence of Modic changes was 22% [13]. In the present study, we found a higher prevalence of Modic changes (56%), which may be explained at least partly by gender difference. Our occupational cohort consisted only of males and a recent study observed that all Modic changes associated significantly with male gender [19]. The high prevalence of Modic changes in the current study compared to the Danish study may also be due to genetic homogeneity, which has been observed for many monogenic diseases in Finland [25], or due to the older age of our population. The prevalence of Modic changes has been found to be associated with age [2,19,20]. Similarly, we found a close association between age and the prevalence of changes as an evidence of their degenerative etiology. In addition, Modic changes most likely occurred at L4-5 and L5-S1.

In the present study, Modic changes were associated with weight-related factors (BMI, waist circumference). However, these factors were not related to disc degeneration. In another series of patients, Modic changes were associated with increasing weight, but not with BMI [19]. Obesity has been suggested as a risk factor for disc degeneration, but the results remains controversial [7-10].

In this study there was a significant association between lifetime leisure exercise activity and all Modic changes (type I and II combined) at L5-S1 in the multivariate analyses (data not shown). When types I and II were analyzed separately, no significant associations remained (Table 3). There is only limited knowledge about the association of physical activity and Modic changes. One population-based study observed that the prevalence of high-level physical activity at leisure time did not differ between subjects with Modic changes and disc degeneration and those with only disc degeneration [14]. However, in the Danish study occupational physical load was significantly higher in the subgroup with Modic changes and disc degeneration in comparison with disc degeneration but without Modic changes, whereas our study subjects had non-physical jobs. Videman et al. [12] detected a relation between vigorous exercise and lumbar degenerative changes among former Finnish elite athletes. However, they did not evaluate Modic changes separately. Recent studies on identical twins have shown that 70% of intervertebral disc degeneration can be attributed to familial factors rather than to the mechanical environment [11]. Endplate is, however, a weak link of the spine in compression, and always fails before the intervertebral disc, even if the latter is injured before loading commences [26]. We speculate that mechanical loading damages the endplate and may lead to activation of the degeneration. This may be first visible in MR images as a more active Modic type I lesion, and if mechanical loading continues, it converts to a more stable type II lesion [2,20].

Smoking has been suspected to carry deleterious effects on the intervertebral discs. According to a systematic review, smoking is associated with LBP [27]. In our study population, there was a trend between smoking (assessed as pack-years) and a higher prevalence of Modic changes (type I and II combined) at L5-S1, but no association with severe disc degeneration. Although an association between smoking and disc degeneration was observed in an earlier study on Finnish twins [28], the association between smoking and disc degeneration was not confirmed in a later study [5]. The study on effect of smoking on Modic changes should be replicated in another population-based cohort. At the moment, we are unable to either disprove or support the hypothesis that smoking is a risk factor for degenerative imaging findings.

Another factor of environmental exposure, alcohol, has been associated with many organ abnormalities and cognitive deficits. However, little is known about the effects of alcohol on the intervertebral discs. We found no association between alcohol consumption and Modic changes or disc degeneration. It therefore seems likely that alcohol does not have deleterious effects on the intervertebral discs.

Occupation (train engineers vs. sedentary factory work) or the duration of exposure to whole-body vibration was of borderline significance for type II Modic changes located at L5-S1. Interestingly, whole-body vibration was associated with severe disc degeneration, but only at L5-S1. There is evidence of an association between vibration and degenerative spinal changes [29] but this could not be confirmed in a high-quality twin study with discordant occupational driving exposure [30]. Although our findings indicate that exposure to whole-body vibration may accelerate disc degeneration (and possibly Modic changes), we cannot wholly exclude a chance finding.

Our results on the determinants of Modic changes are relevant as recent publications suggest that Modic changes are associated with low back symptoms [13,14,21]. Furthermore, changes located at L5-S1 and Modic type I lesions may be more likely to be associated with pain symptoms than other types of Modic changes or changes located at other lumbar levels [17]. In this context it is interesting that the determinants of Modic changes and severe disc degeneration differ especially at L5-S1 level.

The strength of our study is based on the occupational cohort consisting of train engineers and sedentary factory workers of similar age range. Additionally, all train engineers were full-time train drivers from the northern Finland, which ensured a very similar exposure to whole-body vibration. The factory workers consisted of paper mill and chemical factory workers from the same area with only sedentary job and no vibration exposure.

Limitations of our study are based on its cross-sectional nature, which is a common problem in epidemiological studies. Even if selected determinants can be reasonably well assessed retrospectively, their temporal relation to the development of Modic changes or disc degeneration can not be demonstrated. The effect of age, and maybe of genetic factors, may also dilute the effect of environmental determinants. Additionally, especially the assessment of lifetime leisure exercise is susceptible to recall bias. The validity of the original questionnaire has been well documented with respect to cardiovascular outcomes [22]. However, it estimates caloric expenditure more than weight bearing or strength-related dimensions of physical activity. Thus, its sensitivity to detect dimensions of physical activity may not be optimal. Furthermore, we did not have precise measurements of whole-body vibration exposure. When studying lifetime effects, such precision is not feasible due to variations in train models and railway conditions.

Based on the findings of our study, the determinants of Modic type I and II changes are different. The determinants of Type I remain unidentified, whereas age and weight-related factors (BMI, waist circumference) were related to type II changes. This may be partly explained by the low number of subjects with type I changes. However, type I changes are also associated with an ongoing degenerative process which over time converts to Type II or normal bone marrow [21]. Finally, the influence of determinants may also vary at different stages of the degenerative process.

## Conclusion

Weight-related factors, which add to the load of the lumbar spine, seem important in the pathogenesis of Modic changes, whereas whole-body vibration does not have an effect. Conversely, weight-related factors were not associated with severe disc degeneration, whereas whole-body vibration associated with severe disc degeneration at L5-S1.

## Competing interests

The author(s) declare that they have no competing interests.

## Authors' contributions

MK, JK, JN and OT conceived and designed the study protocol. RK, KK and AN participated in the design and coordination of the study. MK, JN and RO analysed the MRI data. MK, JK, MHa, MHe, ST and OT were involved in interpretation of the results. MHa and MHe designed the statistical analysis. MK drafted the manuscript and JK, MHa, JN, RO, MHe, RK, KK, ST, AN and OT contributed to the manuscript. All authors read and approved the final manuscript.

## Pre-publication history

The pre-publication history for this paper can be accessed here:


